# Value and Kinetics of Virological Markers in the Natural Course of Chronic Hepatitis D Virus Infection

**DOI:** 10.1111/liv.70003

**Published:** 2025-01-23

**Authors:** Lisa Sandmann, Valerie Ohlendorf, Alena Ehrenbauer, Birgit Bremer, Anke R. M. Kraft, Markus Cornberg, Katja Deterding, Heiner Wedemeyer, Benjamin Maasoumy

**Affiliations:** ^1^ Department of Gastroenterology, Hepatology, Infectious Diseases and Endocrinology Hannover Medical School Hanover Germany; ^2^ D‐SOLVE Consortium, an EU Horizon Europe Funded Project partner site Hannover Germany; ^3^ Cluster of Excellence RESIST (EXC 2155) Hannover Medical School Hannover Germany; ^4^ German Center for Infection Research (DZIF) Hannover/Braunschweig Germany; ^5^ Centre for Individualised Infection Medicine (CiiM), a Joint Venture Between Helmholtz‐Centre for Infection Research and Hannover Medical School Hannover Germany

**Keywords:** anti‐HBc, HBcrAg, HBV RNA, HBV/HDV coinfection, outcome

## Abstract

**Background and Aims:**

Chronic hepatitis D virus (HDV) infection can cause severe liver disease. With new treatment options available, it is important to identify patients at risk for liver‐related complications. We aimed to investigate kinetics and predictive values of novel virological and immunological markers in the natural course of chronic HDV infection.

**Methods:**

HBcrAg, HBV RNA and quantitative anti‐HBc were analysed in samples from HDV‐infected patients at three consecutive time points. Results were linked to clinical outcome by univariable and multivariable analyses. Primary endpoint was the composite endpoint of any liver‐related event.

**Results:**

Samples from 190 individual patients were analysed with a median clinical follow‐up time of 2.69 (IQR 1.13–6.51) years. The majority of patients had cirrhosis (98/190, 52%), and the primary endpoint occurred in 33% (62/190). In univariable analysis, age, cirrhosis, lower quantitative anti‐HBc, higher ratio of HBcrAg/anti‐HBc and detectable HDV RNA were associated with the primary endpoint. In multivariable analysis, only the presence of liver cirrhosis (HR 7.74, *p* < 0.001) and age (1.06, p < 0.001) remained independently associated with the primary endpoint. Kinetics of virological parameters during follow‐up were similar between the groups. Quantitative anti‐HBc was significantly lower in patients with liver cirrhosis (687 (IQR 188–3388) IU/ml vs. 309 (IQR 82–924) IU/ml, *p* < 0.0004), and lower levels were independently associated with the development of the primary endpoint (HR 1.0, *p* = 0.014).

**Conclusion:**

In chronic HDV infection, neither baseline values nor kinetics of HBV RNA, HBcrAg and anti‐HBc were independently associated with clinical outcome, while stage of liver disease and age were predictors of liver‐related events.

**Abbreviations**
ALT: alanine aminotransferase
AP: alkaline phosphatase
AST: aspartate aminotransferase
BL: baseline
BLV: bulevirtide
CHE: cholinesterase
CI: confidence interval
FU1: follow‐up 1
FU2: follow‐up 2
HBc: hepatitis B core
HBcrAg: hepatitis B core–related antigen
HBsAg: hepatitis B surface antigen
HBV: hepatitis B virus
HCC: hepatocellular carcinoma
HDV: hepatitis D virus
HR: hazard ratio
INR: international normalized ratio
IQR: interquartile range
LLOD: lower limit of detection
LLOQ: lower limit of quantification
NA: nucleos(t)ide analogues
PEG‐IFNa: pegylated interferon alfa

**Summary**

- In this well‐characterized cohort of 190 HDV‐infected patients with a long follow‐up, neither baseline levels nor kinetics of HBcrAg, HBV RNA or quantitative anti‐HBc were independently associated with clinical outcome while stage of liver disease and age were predictors of liver‐related events.
- Interestingly, quantitative anti‐HBc was significantly lower in patients with liver cirrhosis and especially in those developing liver‐related endpoints.
- This encourages further research, particularly in the context of antiviral treatment that aims to achieve immunological control.

## 1 Introduction

Chronic hepatitis D virus (HDV) infection is a rare but relevant disease as it leads to liver cirrhosis and associated complications such as hepatic decompensation, development of hepatocellular carcinoma (HCC) and liver‐related death [1]. Antiviral treatment with pegylated interferon alfa (PEG‐IFNa) or bulevirtide (BLV) is available, but treatment uptake is affected by various barriers, for example limited availability, costs, side effects or stage of liver disease. Parameters to predict the natural course of chronic HDV infection are important to identify patients at risk of developing complications and prioritize patients for antiviral treatment. Virological parameters such as HDV RNA or anti‐HDV IgM have been proposed for the prediction of disease activity and risk of disease progression: the association between HDV RNA status and liver‐related morbidity and mortality was investigated in a meta‐analysis [2]. In this analysis, patients with detectable HDV RNA had a higher risk of liver‐related complications, including hepatic decompensation, development of HCC and liver‐related death. The presence of anti‐HDV IgM antibodies has been associated with the development of liver‐related endpoints during a median follow‐up of 3 years [3]. New virological and immunological markers such as hepatitis B core–related antigen (HBcrAg), HBV RNA and quantitative anti‐HBc have been evaluated in the context of chronic hepatitis B virus (HBV) infection [4, 5, 6, 7, 8], but data in chronic HDV infection are limited. It has been shown that during antiviral treatment, HBcrAg levels can serve as an additional marker to predict treatment response [9, 10]. However, kinetics and predictive value of these markers in the natural course of chronic HDV infection and the potential link to the risk for liver‐related events are unknown. The aim of this study is to investigate novel virological and immunological markers, namely HBcrAg, HBV RNA and quantitative anti‐HBc in the natural course of chronic HDV infection in a well‐characterized, large clinical cohort of HDV‐infected patients.

## 2 Methods

### 2.1 Study Cohort

Consecutive patients with HDV infection and available serum samples were identified retrospectively at Hannover Medical School. Chronic HDV infection was defined as HBsAg and anti‐HDV positivity or detectable HDV RNA for at least 6 months. Clinical data were collected from medical records. Patients with replicative hepatitis C virus or human immunodeficiency virus infection were excluded. Baseline (BL) was defined as the earliest clinical visit with an available serum sample in the absence of HDV‐directed therapy (PEG‐IFNa or BLV). Treatment with nucleos(t)ide analogues (NA) was not an exclusion criterion. Two additional follow‐up time points were chosen for virological analyses: 6 months (±3 months) after BL (FU1) and 2 to 4 years (±6 months) after BL (FU2). Importantly, treatment conditions had to be similar at and in between all time points to exclude treatment effects. Samples for follow‐up analyses were excluded if treatment with PEG‐IFNa, BLV or NA was started between BL and the respective follow‐up time point. Liver cirrhosis at BL was defined based on liver histology if available, ultrasound findings typical for cirrhosis or transient elastography > 15 kPa. If these data were not available, the presence of cirrhosis was considered if patients had already clinical evidence of hepatic decompensation in the past or if at least two of the following criteria were present: platelets < 100 000/mL, international normalized ratio (INR) > 1.5, presence of oesophageal varices and/or splenomegaly (largest dimension > 13 cm).

### 2.2 Definition of Endpoints

Clinical outcome was assessed at the last clinical visit of the patient. If HDV‐directed treatment was initiated during follow‐up, the last visit before treatment initiation was selected to evaluate clinical outcome. The primary endpoint was defined as the composite endpoint of liver‐related events including hepatic decompensation (ascites, hepatic encephalopathy, variceal bleeding), HCC, liver transplantation or liver‐related death. Secondary endpoints were hepatic decompensation and/or HCC, liver transplantation and/or liver‐related death and HBsAg loss.

### 2.3 Laboratory Testing

Serum levels of HDV RNA, HBV RNA, HBcrAg and anti‐HBc were measured at BL and the respective follow‐up time points. HBV RNA was measured using the Roche Cobas 6800 platform with a lower limit of quantification (LLOQ) of 10 cp/ml, HBcrAg by using the Lumipulse G HBcrAg Immunoreaction assay (Lumipulse G Fujirebio‐Europe (LLOQ 3 log U/ml)) and anti‐HBc by using the Lumipulse G HBcAb‐N Immunoreaction assay (Lumipulse G Fujirebio‐Europe (LLOQ 1 IU/mL)). The lower limit of detection (LLOD) of the HBcrAg assay is 2 log U/ml. For dichotomous analyses, HBV RNA and HBcrAg levels below the LLOQ were regarded as ‘undetectable’. For quantification of HDV RNA, the RoboGene HDV RNA Quantification Kit 2.0 was used (RoboGene GmbH, LLOQ 82 IU/mL). Routine laboratory parameters were determined at the central laboratory as part of the clinical work‐up.

### 2.4 Statistical Analysis

Statistical analyses were performed by using SPSS statistics version 28 (IBM Corp. Released 2021. IBM SPSS Statistics for Windows, Version 28.0. Armonk, NY: IBM Corp), GraphPad Prism version 10.2.1 for Windows (GraphPad Software, San Diego, California USA) and R (Version 4.2.0; packages ‘cmprsk’, ‘RCmdr’ and ‘RcmdrPlugin.EZR’ [11]). Detailed information on the statistical analyses is provided in the supplement.

### 2.5 Ethics Approval and Patient Consent Statement

Patients from the prospective registry provided written informed consent. Informed consent was waived for the analyses of archived samples and retrospectively collected clinical data. The study was approved by the ethics committee of Hannover Medical School (No. 9356_BO_K_2020, No. 9227_BO_K_2020) and was carried out in accordance with the Declaration of Helsinki and Istanbul.

## 3 Results

### 3.1 Study Cohort

A total of 190 patients with available BL samples were identified. More than half of the patients were classified as having liver cirrhosis (52%, 98/190). HDV RNA, HBcrAg and HBV RNA were detectable in 84%, 74% and 11% of patients, respectively. NA treatment was present in 43% (82/190) of patients and 35% (67/190) received IFN treatment in the past. Detailed baseline characteristics are depicted in Table 1.

**TABLE 1:** Baseline characteristics. Continuous parameters are depicted as median with interquartile range, categorical variables as number with percentage.

Total, *n*	190
Male, *n* (%)	124 (65)
Age, years	41.3 (32.4–49.7)
HDV RNA (log_10_ IU/mL)	4.3 (2.55–5.54)
HDV RNA undetectable, *n* (%)	31 (16)
HBcrAg (log_10_ U/mL)	3.9 (2.9–4.73)
HBcrAg ≥ 3 log_10_ U/mL, *n* (%)	141 (74)
HBcrAg < 3 log_10_ U/mL or undetectable, *n* (%)	49 (26)
HBV RNA^a^	
HBV RNA ≥ 10 cop/mL, *n* (%)	20 (11)
HBV RNA < 10 cop/ml or undetectable, *n* (%)	155 (89)
Anti‐HBc (IU/mL)	469 (123.5–1570)
HBV DNA (IU/mL)	20 (0–58.2)
HBsAg (IU/mL)	8490 (2250–14 032)
HBeAg positive, *n* (%)^b^	25 (14)
ALT (U/L)	64 (38–132)
AST (U/L)	64 (40–94)
Platelets (x1000/μl)	135 (64–184.3)
Cirrhosis, *n* (%)	98 (52)
NA treatment, *n* (%)	82 (43)
Previous IFN treatment, *n* (%)	67 (35)

Abbreviations: ALT, alanine aminotransferase; AST, aspartate aminotransferase; IFN, interferon; NA, nucleos(t)ide analogue.

^a^ Available for 175 patients.

^b^ Available for 183 patients.

### 3.2 Kinetics of HDV RNA, HBsAg, HBV RNA, HBcrAg and Anti‐HBc in the Natural Course of Chronic HDV Infection

**FIGURE 1:**
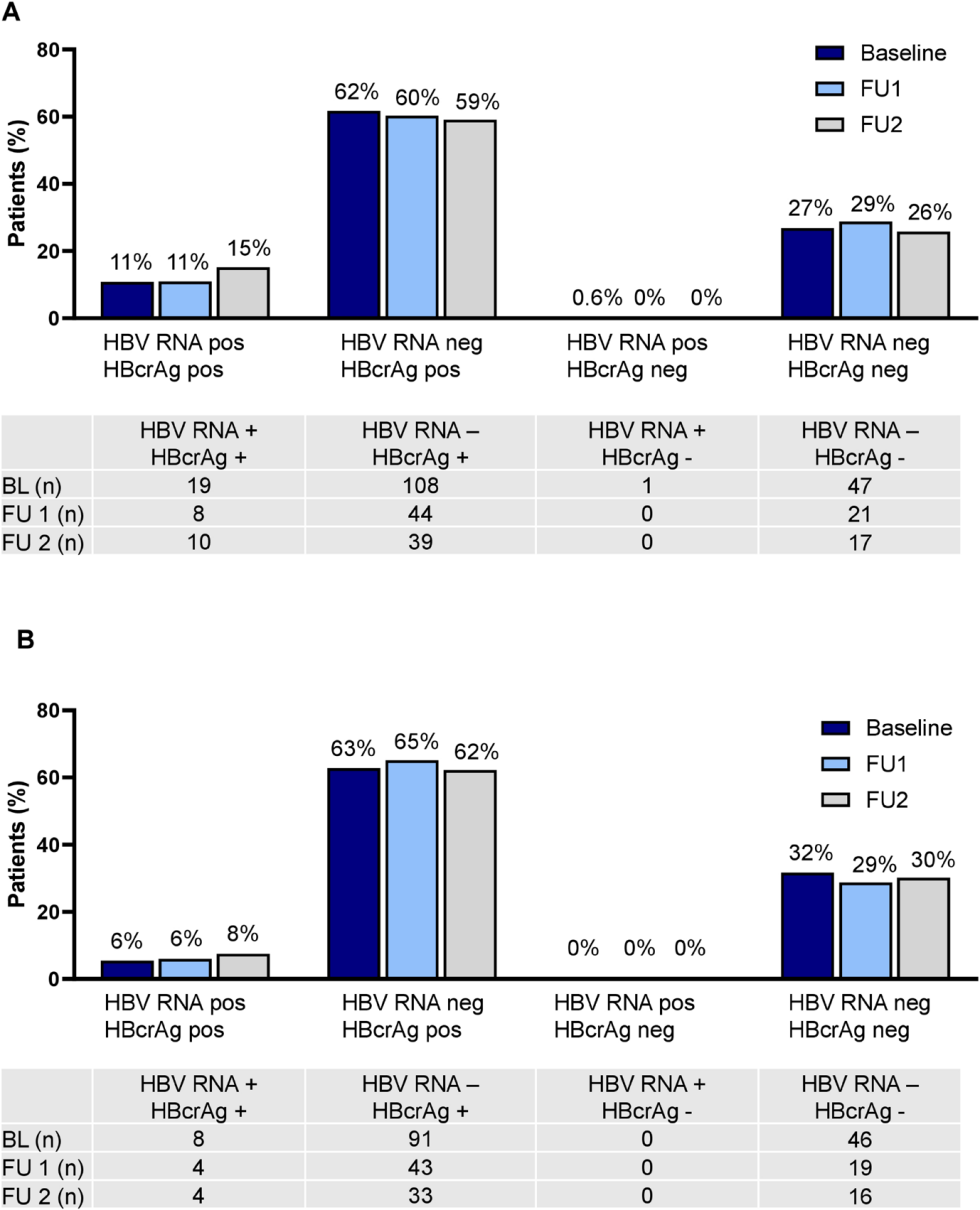
Proportion of all (A) or HBeAg‐negative (B) patients with concordant or discordant levels of HBcrAg or HBV RNA at study time points. Undetectable HBcrAg is defined as HBcrAg < 3 U/mL, and undetectable HBV RNA is defined as HBV RNA < 10 IU/ml.

Based on the predefined follow‐up criteria, 73, 66 and 44 patients were identified for FU1, FU2 and FU1/FU2 analyses (Table 2). Median time from BL to FU1 or FU2 was 5.8 (IQR 4.3–6.7) and 27.3 (24.4–34.3) months, respectively. From BL to FU1 and FU2, median levels of HBcrAg and anti‐HBc declined significantly, while no changes were detected for HDV RNA, HBV RNA or HBsAg levels. From BL to FU2, an additional HDV RNA decline was detected (4.29 log_10_ IU/ml (2.04–5.2 log_10_ IU/ml) vs. 3.29 log_10_ IU/ml (1.27–5.12 log_10_ IU/ml), *p* = 0.0041), while other findings were comparable to the short follow‐up period (Table 2). HBV RNA levels were low at all study time points with only a minority of samples showing detectable HBV RNA levels. No significant differences were present in the proportion of samples with concordant detectable/undetectable or discordant detectable/undetectable HBcrAg and HBV RNA levels at all study time points. The majority of samples had detectable HBcrAg and undetectable HBV RNA levels (Figure 1A). There were no significant differences when only HBeAg‐negative patients were included (Figure 1B and Table S1).

**TABLE 2:** Comparison of median levels of virological parameters at the respective study time points. Median levels with interquartile range are depicted. Wilcoxon signed‐rank test was used for comparison of medians.

	Baseline	Follow‐up 1	*p* value	Follow‐up 2	*p* value
*n* = 73					
HDV RNA (log_10_ IU/mL)	4.36 (1.44–5.2)	3.86 (0–5.48)	0.1010		
HBcrAg (log_10_ U/mL)	4.0 (2.85–4.60)	3.9 (2.0–4.65)	**0.0142**		
HBV RNA (cop/ml)	0 (0–1)	0 (0–1)	0.3026		
Anti‐HBc (IU/mL)	474 (69–1870)	332 (58–1505)	**0.0018**		
HBsAg (IU/mL)	6549 (1788–14 025)	5679 (1613–12 630)	0.2119		
*n* = 66					
HDV RNA (log_10_ IU/ml)	4.29 (2.04–5.2)			3.29 (1.27–5.12)	**0.0041**
HBcrAg (log_10_ U/ml)	4.25 (3.0–5.1)			3.9 (2.68–4.9)	**0.0003**
HBV RNA (cop/ml)	0 (0–1)			0 (0–1)	0.0984
Anti‐HBc (IU/ml)	553 (127–1815)			310 (70–1073)	< **0.0001**
HBsAg (IU/ml)	7899 (2490–14 177)			10 069 (4213–18 572)	0.2424
*n* = 44					
HDV RNA (log_10_ IU/ml)	4.29 (1.89–5.25)	3.87 (1.15–5.69)	0.1607	3.59 (1.27–5.21)	0.0740
HBcrAg (log_10_ U/ml)	4.3 (3.13–4.8)	4.2 (3.1–4.88)	**0.0324**	4.05 (3.0–4.7)	**0.0035**
HBV RNA (cop/ml)	0 (0–1)	0 (0–1)	0.0871	0 (0–0.75)	0.0816
Anti‐HBc (IU/ml)	444 (88–1640)	288 (64–1428)	0.0633	215 (57–580)	**0.0003**
HBsAg (IU/ml)	9651 (2355–14 291)	9486 (2798–14 908)	0.6420	6284 (1620–13 024)	0.2059

*Note:* bold indicates *p* values < 0.05.

### 3.3 Correlation of Virological Parameters

Significant positive correlations between HBcrAg and HBV RNA, HDV RNA and HBsAg were present at all study time points irrespective of HBeAg status (Table 3, Figures S1 and S2, Table S2). No relevant differences were detected when separating according to the presence or absence of NA treatment (Table S3).

**TABLE 3:** Correlation of virological parameters at baseline, after 6 months (follow‐up 1) and 2–4 years (follow‐up 2) of follow‐up. Spearman correlation was used to calculate correlations.

		HBV RNA	HBcrAg	Anti‐HBc	HDV RNA
Baseline	HBcrAg	0.363**			
	Anti‐HBc	0.349**	0.1397		
	HDV RNA	0.132	0.305**	−0.179*	
	HBsAg	0.023	0.523**	−0.056	0.519**
Follow‐up 1	HBcrAg	0.376**			
	Anti‐HBc	0.308**	0.026		
	HDV RNA	0.158	0.410**	−0.127	
	HBsAg	0.146	0.662**	0.060	0.631**
Follow‐up 2	HBcrAg	0.434**			
	Anti‐HBc	0.232	0.045		
	HDV RNA	0.228	0.464**	−0.109	
	HBsAg	0.089	0.576**	−0.024	0.730**

* *p* < 0.05.

** *p* < 0.001.

### 3.4 Association of Virological Parameters and Kinetics With Clinical Outcome

Median follow‐up time for the evaluation of the endpoints was 2.69 (IQR 1.13–6.51) years with a maximum follow‐up of 24.5 years. The primary, combined endpoint occurred in 33% (62/190) of patients after a median follow‐up of 1.56 (IQR 0.26–3.37) years. At BL, patients with future endpoint development were significantly older (50.1 (IQR 41.4–57.0) years vs. 38.1 (29.1–44.8) years, *p* < 0.001) and showed a significantly higher frequency of liver cirrhosis (90% vs. 33%, *p* < 0.001) (Table 4). These patients had significantly lower levels of quantitative anti‐HBc, higher ratio of HBcrAg/anti‐HBc and a higher proportion of detectable HDV RNA. In the multivariable cox regression analyses, only liver cirrhosis (HR 7.52, 95% CI 3.18–17.77, p < 0.001) and older age (HR 1.06, 95% CI 1.04–1.09, p < 0.001) remained independently associated with the development of the primary endpoint. Kinetics of HBcrAg, anti‐HBc and HDV RNA from BL until FU2 were not significantly different between patients with and without the development of the primary endpoint (Table S4).

**TABLE 4:** Uni‐ and multivariate analysis of baseline characteristics of patients with and without the development of the combined endpoint (decompensation, HCC, LTx/death) during follow‐up. Continuous parameters are depicted as median with interquartile range, categorical variables as number with percentage. Mann Whitney U test, Chi‐Square or Fisher's exact test were used for group comparison. Multivariable Cox regression was used to address independent association of variables with the development of the combined endpoint during follow‐up.

	Development of the combined endpoint			Multivariable analysis: Model A			Multivariable analysis: Model B		
	No (128)	Yes (62)	*p* value	HR	95% CI	*p* value	HR	95% CI	*p* value
Male, *n* (%)	79 (62)	45 (73)	0.140						
Age, years	38.1 (29.1–44.8)	50.1 (41.4–57.0)	**< 0.001**	1.064	1.036–1.094	**< 0.001**	1.063	1.034–1.092	**< 0.001**
Cirrhosis, *n* (%)	42 (33)	56 (90)	**< 0.001**	7.739	3.239–18.49	**< 0.001**	7.355	3.112–17.38	**< 0.001**
NA treatment	50 (39)	32 (52)	0.101						
IFN prior to BL	47 (37)	20 (32)	0.546						
HBV RNA detectable^a^	17 (14)	3 (6)	0.093						
HBcrAg (log_10_ U/mL)	3.85 (2.63–4.7)	3.95 (3.08–4.8)	0.459						
HBcrAg detectable	93 (73)	48 (77)	0.482						
Anti‐HBc (IU/mL)	587 (176–2246)	214 (51–667)	**< 0.001**	1.0	1.0–1.0	0.3341			
HBcrAg/anti‐HBc ratio	1.40 (0.97–1.85)	1.69 (1.24–2.31)	**0.002**				1.096	0.8444–1.423	0.4899
HBsAg (IU/mL)^b^	8628 (2114–14 071)	7016 (2250–12 636)	0.593						
HDV RNA (log_10_ IU/mL)	4.19 (1.15–5.56)	4.78 (3.26–5.54)	0.184						
HDV RNA detectable	102 (80)	57 (92)	**0.032**	2.094	0.7928–5.532	0.1358	1.747	0.6845–4.461	0.2431

Abbreviations: CI, confidence interval; HR, hazard ratio; IFN, interferon; NA, nucleos(t)ide analogue.

^a^ Available for 120 and 55, respectively.

^b^ Available for 92 and 40, respectively.

*Note:* bold indicates *p* values < 0.05.

Comparable results were obtained for the secondary endpoints. Virological parameters at BL were not independently associated with the development of death or liver transplantation (*n* = 39; Table S5), hepatic decompensation (*n* = 40) and HCC (*n* = 55; Table S6) or HCC alone (*n* = 23; Table S7).

During the follow‐up period, nine patients showed HBsAg loss. Levels of HBcrAg, HBsAg and HDV RNA were significantly lower in patients with future HBsAg loss (Table S8). Due to the low number of events, no multivariable competing risk analysis was performed.

To further elucidate the role of qualitative HDV RNA, the cohort was separated based on HDV RNA status at BL. Patients with detectable HDV RNA and the development of the combined endpoint were significantly older (49.7 (41.2–55‐1) years vs. (37.7 (29.1–44.6) years, *p* < 0.001)), showed a higher frequency of liver cirrhosis (89.5% vs. 33.3%, *p* < 0.001) and lower levels of quantitative anti‐HBc along with a higher ratio of HBcrAg/anti‐HBc. In the multivariable cox regression analyses, only cirrhosis (HR 6.33, 95% CI 2.64–15.13, *p* < 0.001) and age (HR 1.06, 95% CI 1.03–1.09, *p* < 0.001) remained independently associated with the development of the primary endpoint (Table S9). For patients with undetectable HDV RNA, also older age and presence of liver cirrhosis were associated with the development of the combined endpoint (Table S10). Multivariate analysis was not possible due to the low number of events (*n* = 6) in this subgroup.

### 3.5 Virological Parameters in Patients With Liver Cirrhosis

At BL, the majority of patients was diagnosed with liver cirrhosis. Patients with liver cirrhosis were significantly older (46.2 (38–52.4) vs. 36.3 (27.6–42.9) years, *p* < 0.001) and were more likely to receive NA treatment (50% vs. 36%, *p* = 0.049) (Table S11). They showed higher proportions of detectable HBV RNA levels and significantly lower levels of quantitative anti‐HBc (309 (82–924) vs. 687 (188–3388) IU/ml, *p* = 0.0004). No significant differences were detected for HDV RNA, HBcrAg or HBsAg levels. The subgroup of patients with liver cirrhosis and the development of the primary endpoint were significantly older and had more advanced liver disease as reflected by significantly lower platelet and albumin levels and higher levels of bilirubin (Table 5). Interestingly, also levels of anti‐HBc were significantly lower in patients with liver cirrhosis and the development of the primary endpoint. In the multivariable cox regression analysis, all parameters remained independently associated with the development of the primary endpoint in the subgroup of patients with liver cirrhosis.

**TABLE 5:** Comparison of baseline characteristics of patients with liver cirrhosis with and without the development of the combined endpoint during follow‐up. Continuous parameters are depicted as median with interquartile range, categorical variables as number with percentage. Mann Whitney *U* test, chi‐Square or Fisher's exact test were used for group comparison. Multivariable analysis was performed by cox regression analysis. Due to the strong baseline correlation between INR and CHE (0.639), and CHE and albumin (0.677) only CHE was included in the multivariable model.

	Development of the combined endpoint			Multivariable analysis		
	No (*n* = 42)	Yes (*n* = 56)	*p* value	Hazard ratio	95% CI	*p* value
Male, *n* (%)	29 (69)	40 (71)	0.798			
Age, years	41.5 (33.2–47.6)	50.1 (41.4–55.3)	**< 0.001**	1.046	1.008–1.087	0.019
NA treatment	20 (48)	29 (52)	0.683			
IFN prior to BL	14 (33)	18 (32)	0.901			
HBV RNA detectable^a^	3 (7)	2 (4)	0.654			
HBcrAg (log_10_ U/ml)	3.8 (3.0–4.6)	3.85 (3.03–4.68)	0.752			
HBcrAg detectable	33 (79)	43 (77)	0.834			
Anti‐HBc (IU/ml)	433 (127–1335)	214 (51–644)	**0.047**	1.0	1.0–1.0	0.014
HBcrAg/anti‐HBc ratio	1.45 (1.11–1.95)	1.67 (1.23–2.28)	0.102			
HBsAg (IU/ml)^b^	88 818 (1419–11 756)	7016 (2250–12 608)	0.835			
HDV RNA (log_10_ IU/mL)	4.02 (1.53–5.46)	4.63 (3.01–5.46)	0.309			
HDV RNA detectable	34 (81)	51 (91)	0.144			
Sodium mmol/L	140 (137–141)	139 (137–141)	0.639			
Creatinine μmol/L	69 (59–80)	66 (55–74)	0.117			
AST U/L	63 (38–89)	80 (56–105)	**0.015**	1.009	1.004–1.014	< 0.001
ALT U/L	58 (36–117)	57 (38–105)	1.0			
gGT U/L	74 (36–159)	71 (35–129)	0.909			
AP U/L	94 (69–128)	143 (106–172)	**< 0.001**	1.005	1.001–1.010	0.011
CHE kU/L	5.19 (3.95–6.91)	3.47 (2.48–4.45)	**< 0.001**	0.483	0.361–0.646	< 0.001
Bilirubin mmol/L	11 (9–19)	20 (16–46)	**< 0.001**	1.006	1.0–1.012	0.043
Albumin g/L	40 (36–41)	33 (28–36)	**< 0.001**			
Platelets x1000/μl	84 (49.5–146.5)	58 (48–96)	**0.023**	1.0	1.0–1.0	0.021
INR	1.17 (1.09–1.26)	1.4 (1.23–1.63)	**< 0.001**			

Abbreviations: ALT, alanine aminotransferase; AP, alkaline phosphatase; AST, aspartate aminotransferase; BL, baseline; CHE, cholinesterase; GGT, γ‐glutamyltransferase; IFN, interferon; INR, international normalized ratio; NA, nucleos(t)ide analogue.

^a^ Available for 91 samples.

^b^ Available for 68 samples.

*Note:* bold indicates *p* values < 0.05.

In the subgroup of patients without liver cirrhosis (*n* = 92), only six patients developed the combined endpoint during a median follow‐up time of 3.74 (IQR 1.22–7.42) years. These patients were significantly older, showed higher levels of quantitative HDV RNA and more advanced liver disease as reflected by lower levels of platelets and albumin and higher INR (Table S12). Due to the low number of events, multivariable competing risk analysis was not performed.

## 4 Discussion

In this study, we provide a comprehensive analysis of novel virological and immunological parameters in a large and well‐characterized cohort of HDV‐infected patients with long‐term follow‐up data. We showed that in the natural course of HBV/HDV coinfection, levels of HDV RNA, HBcrAg and anti‐HBc declined significantly, while HBV RNA levels remained undetectable in the majority of patients. Neither baseline parameters nor kinetics during follow‐up were associated with the development of liver‐related endpoints. Interestingly, quantitative levels of anti‐HBc were significantly lower in the subgroup of patients with liver cirrhosis.

Treatment options for chronic HDV infection are limited to various reasons, that is access to treatment, stage of liver disease or treatment costs. Therefore, parameters for the prediction of disease outcome are needed to identify patients who should be prioritized for treatment. The association between HDV RNA status and liver‐related morbidity and mortality has been widely discussed and recently investigated in a meta‐analysis including 12 studies and 4876 patients [2]. In this study, patients with detectable HDV RNA experienced a higher risk of developing hepatic decompensation and HCC, as well as liver transplantation and death. This is in contrast to our study in which neither HDV RNA levels nor qualitative HDV RNA results were independently associated with the primary or secondary endpoints. In our study, the presence of cirrhosis and age were identified as parameters independently associated with the combined endpoint of hepatic decompensation, HCC, liver transplantation and death. In the abovementioned meta‐analysis, these parameters were only incompletely assessed and only provided by a minority of studies [12]. Still, when reported, effect estimates for HDV RNA positivity were generally lower in studies including ≥ 50% of patients with cirrhosis [2]. This is in line with our study, in which more than half of the patients were classified as having cirrhosis at baseline. In these patients, it is likely that the risk factor of liver cirrhosis outweighs the impact of viremia on clinical outcomes and therefore explains the lesser impact of HDV RNA in our cohort. In line with this, in our cohort significantly higher HDV RNA levels were observed in the subgroup of patients without liver cirrhosis who developed the combined endpoint compared to those who did not.

Interestingly, total quantitative anti‐HBc was identified to be significantly different in patients with and without liver cirrhosis. To our knowledge, only few studies investigated the role of quantitative anti‐HBc in chronic HDV infection [13, 14]. Compared to chronic HBV infection, levels of quantitative anti‐HBc were not significantly different in HBsAg carriers without HBV‐induced liver damage (HBeAg‐positive and HBeAg‐negative chronic HBV infection) and HDV coinfection, whereas significantly higher levels were detected in patients with untreated HBeAg‐negative chronic hepatitis B [13]. For chronic HBV infection, levels of anti‐HBc have been associated with the phase of disease with a positive correlation to ALT levels [13, 15]. Additionally, a positive correlation of quantitative anti‐HBc and fibrosis stage was described for patients with chronic HBV infection [16]. In contrast to this, for chronic HDV infection, total anti‐HBc levels were numerically lower in patients with cirrhosis compared to patients without [14]. This is in line with the results from our cohort showing significantly lower levels of anti‐HBc in patients with liver cirrhosis. Furthermore, we did not detect a significant correlation between quantitative anti‐HBc and ALT levels, which is consistent with own data [9] and the results by Ricco et al. showing lower levels of anti‐HBc in patients with elevated ALT levels. These findings suggest that higher levels of quantitative anti‐HBc are associated with HBV‐related hepatitis, which might be present to a lower extend in chronic HDV infection and advanced HDV‐related liver disease.

As for HBV RNA, only a minority of patients showed detectable HBV RNA levels, which is similar to other cohorts from our centre [9, 17]. In the subgroup of patients with detectable HBV RNA, a strong correlation with HBcrAg was observed. However, neither presence nor absence of detectable HBV RNA was independently associated with the development of liver‐related endpoints. The diagnostic utility of HBV RNA has been intensively discussed in chronic HBV infection [7, 18, 19, 20]. For HDV infection, it has been shown that undetectable levels were associated with response to interferon treatment [17], but due to the low proportion of HBV RNA detectability its diagnostic and prognostic value in chronic HDV infection remains debatable. In chronic HDV infection, the main origin of HBsAg is integrated HBV DNA, which might explain the overall low proportion of detectable HBV RNA that is reflecting HBV cccDNA transcriptional activity [21].

Our study has important limitations. Due to the retrospective design, only patients with available serum samples were included. Therefore, selection bias might influence the results presented in this study. Furthermore, our cohort derives from one single‐centre, university hospital. It has been shown that patients with chronic HDV infection at tertiary centres are likely to present with more advanced liver disease compared to secondary care centres [22], which leads to the risk of overestimating the prevalence of liver‐related endpoints. Still, 48% of patients of our cohort were diagnosed without liver cirrhosis, which results in a well‐balanced cohort of patients with and without advanced liver disease. Along this, the follow‐up time can be seen as a limitation of this study. The median follow‐up duration in our cohort was 2.69 (IQR 1.13–6.51) years, which might be too short to address outcome events in patients without liver cirrhosis. However, in the subgroup of patients without liver cirrhosis, median follow‐up time was 3.75 (IQR 1.22–7.42) years and numerically longer compared to the subgroup of patients with liver cirrhosis (2.40 years (IQR 1.08–5.45)). Lastly, we were not able to investigate HDV genotype or additional virological parameters, that is, HBsAg protein composition or anti‐HDV IgM. Due to the retrospective design of the study, sample volume was limited and required prioritization for virological analyses, such as HDV RNA quantification with the same assay.

To summarize, our study provides important information on the prognostic utility of virological and immunological markers in the natural course of chronic HDV infection. In this well‐characterized cohort, neither baseline values nor kinetics were independently associated with clinical outcome, while stage of liver disease and age were predictors of liver‐related events. Quantitative anti‐HBc could serve as an additional marker to identify HDV‐infected patients with advanced liver disease at risk of developing liver‐related complications. However, further studies are needed to verify this aspect.

## Author Contributions

Conception and design of the study: Lisa Sandmann, Benjamin Maasoumy; funding acquisition: Benjamin Maasoumy; acquisition of data: Lisa Sandmann, Alena Ehrenbauer, Birgit Bremer, Anke R.M. Kraft, Valerie Ohlendorf, Katja Deterding; data analysis: Lisa Sandmann; interpretation of data: Lisa Sandmann, Valerie Ohlendorf, Markus Cornberg, Heiner Wedemeyer, Benjamin Maasoumy; drafting of the manuscript: Lisa Sandmann; reviewing and editing: all authors; final approval of the manuscript: all authors.

## Conflicts of Interest

Lisa Sandmann reports lecture honoraria and personal fees from Falk Pharma e.V., Gilead and Roche, and travel support from AbbVie. Markus Cornberg reports personal fees from AbbVie, Falk Foundation, Gilead, Janssen‐Cilag, GSK, MSD, Spring Bank and SOBI. Katja Deterding received lecture and personal fees from Gilead, Falk Pharma e.V., AbbVie, MSD/Merck and Alnylam. Heiner Wedemeyer reports grants/research support from AbbVie, Biotest, BMS, Gilead, Merck/MSD, Novartis, Roche and personal fees from Abbott, AbbVie, Altimmune, Biotest, BMS, BTG, Dicerna, Gilead, Janssen, Merck/MSD, MYR GmbH, Novartis, Roche and Siemens. Benjamin Maasoumy served as a speaker and/or advisory board member for AbbVie, Fujirebio, Gilead, Luvos, MSD, Norgine, Roche and W. L. Gore & Associates and received research support from Altona, EWIMED, Fujirebio and Roche. Anke R.M. Kraft, Birgit Bremer, Alena Ehrenbauer and Valerie Ohlendorf have nothing to disclose.

## Supporting information


Data S1:

## Data Availability

Data are available from the corresponding author upon reasonable request.
