# Supplementary material for: Value and Kinetics of Virological Markers in the Natural Course of Chronic Hepatitis D Virus Infection
Source: Liver Int. 2025 Jan 23;45(2):e70003. doi: 10.1111/liv.70003 (PMC11756344; doi:10.1111/liv.70003)
Supplement: Supplementary file 1 — Data S1: [file LIV-45-0-s001.zip › Supplementary material_v1.3_R_clean.docx]

**Supplementary material**

[Supplementary methods: Statistical analysis 2](#_Toc184674510)

[Supplementary table 1 3](#_Toc184674511)

[Supplementary table 2 5](#_Toc184674512)

[Supplementary table 3 6](#_Toc184674513)

[Supplementary table 4 8](#_Toc184674514)

[Supplementary table 5 9](#_Toc184674515)

[Supplementary table 6 11](#_Toc184674516)

[Supplementary table 7 13](#_Toc184674517)

[Supplementary table 8 14](#_Toc184674518)

[Supplementary table 9 15](#_Toc184674519)

[Supplementary table 10 17](#_Toc184674520)

[Supplementary table 11 18](#_Toc184674521)

[Supplementary table 12 20](#_Toc184674522)

[Supplementary figures 22](#_Toc184674523)

[Supplementary figure 1 22](#_Toc184674524)

[Supplementary figure 2 22](#_Toc184674525)

# Supplementary methods: Statistical analysis

Statistical analyses were performed by using SPSS statistics version 28 (IBM Corp. Released 2021. IBM SPSS Statistics for Windows, Version 28.0. Armonk, NY: IBM Corp), GraphPad Prism version 10.2.1 for Windows (GraphPad Software, San Diego, California USA) and R (Version 4.2.0; packages “cmprsk”, “RCmdr” and “RcmdrPlugin.EZR” (10)). Detailed information on the statistical analyses is provided in the supplement. Continuous parameters are presented as median with interquartile range and categorical variables as numbers and percentages. Continuous data were tested for normal distribution using the Shapiro-Wilk test and analyzed using the unpaired t test (normally distributed) or Mann-Whitney U test (not normally distributed). For categorical variables Chi-Square or Fisher’s exact test were used. Differences between HDV RNA, HBV RNA, HBcrAg, anti-HBc at BL and follow-up were calculated by Wilcoxon test for paired testing. Correlation analyses were performed by using Spearman correlation.

For determining the predictive value of different virological and clinical parameters, time-to-event analyses were conducted using Fine and Gray competing risk analysis (11) for the development of hepatic decompensation, HCC or HBsAg-loss or Cox regression for the combined endpoint or liver transplantation/death. For competing risk analyses, death and liver transplantation were considered as competing events. Significant variables from univariable analyses were included in multivariable analyses. P values of <0.05 were considered to be statistically significant.

Supplementary table 1

| n=66 | Baseline | Follow-up 1 | p-value | Follow-up 2 | p-value |
| --- | --- | --- | --- | --- | --- |
| HDV RNA (log_10_ IU/ml) | 4.3 (1.6-5.05) | 3.86 (0-5.1) | 0.0666 |  |  |
| HBcrAg (log_10_ U/ml) | 4.0 (2.88-4.5) | 3.9 (2.0-4.6) | **0.0444** |  |  |
| HBV RNA (cop/ml) | 0 (0-1) | 0 (0-1) | 0.4394 |  |  |
| anti-HBc (IU/ml) | 444 (55-1685) | 309 (47-1555) | **0.0030** |  |  |
| HBsAg (IU/ml) | 6972 (1839-14028) | 6316 (1829-12630) | 0.1711 |  |  |
| n=53 |  |  |  |  |  |
| HDV RNA (log_10_ IU/ml) | 4.26 (1.47-5.1) |  |  | 2.95 (0-5.0) | **0.0088** |
| HBcrAg (log_10_ U/ml) | 3.7 (2.95-4.5) |  |  | 3.6 (2.4-4.3) | **0.0004** |
| HBV RNA (cop/ml) | 0 (0-1) |  |  | 0 (0-0) | 0.4185 |
| anti-HBc (IU/ml) | 505 (110-1621) |  |  | 296 (67-845) | **0.0001** |
| HBsAg (IU/ml) | 9651 (3768-14291) |  |  | 6339 (2098-13408) | 0.3256 |
| n=39 |  |  |  |  |  |
| HDV RNA (log_10_ IU/ml) | 4.29 (1.75-5.2) | 3.85 (1.15-5.34) | 0.1529 | 3.59 (0-5.12) | 0.3309 |
| HBcrAg (log_10_ U/ml) | 4.2 (3.1-4.6) | 4.1 (3.1-4.6) | 0.0645 | 4.0 (3.0-4.6) | 0.1472 |
| HBV RNA (cop/ml) | 0 (0-1) | 0 (0-0) | 0.0578 | 0 (0-0) | 0.9739 |
| anti-HBc (IU/ml) | 413 (56-1581) | 245 (47-1540) | 0.2363 | 245 (47-1540) | **0.0062** |
| HBsAg (IU/ml) | 9860 (2820-14247) | 9651 (3764-14879) | 0.5506 | 6908 (2294-13024) | 0.3246 |

Supplementary table 1. Comparison of median levels of virological parameters at the respective study time points of samples from HBeAg-negative patients. Median levels with interquartile range are depicted. Wilcoxon signed-rank test was used for comparison of medians.

|  |  | HBV RNA | HBcrAg | Anti-HBc | HDV RNA |
| --- | --- | --- | --- | --- | --- |
| Baseline | HBcrAg | 0.189** |  |  |  |
|  | Anti-HBc | 0.358** | 0.022 |  |  |
|  | HDV RNA | 0.029 | 0.251** | -0.237** |  |
|  | HBsAg | -0.025 | 0.480** | -0.131 | 0.556** |
| Follow-up 1 | HBcrAg | 0.234 |  |  |  |
|  | Anti-HBc | 0.252* | -0.063 |  |  |
|  | HDV RNA | 0.080 | 0.330** | -0.180 |  |
|  | HBsAg | 0.148 | 0.652** | 0.045 | 0.599** |
| Follow-up 2 | HBcrAg | 0.190 |  |  |  |
|  | Anti-HBc | 0.227 | -0.194 |  |  |
|  | HDV RNA | 0.129 | 0.474** | -0.161 |  |
|  | HBsAg | 0.077 | 0.628** | -0.092 | 0.765** |

# Supplementary table 2

Supplementary table 2. Correlation of virological parameters at baseline, after 6 months and 2-4 years of follow-up of HBeAg-negative patients. Spearman correlation was used to calculate correlations. * p < 0.05 ** p < 0.001

# Supplementary table 3

|  |  | NA-treatment | | | | No NA-treatment | | | |
| --- | --- | --- | --- | --- | --- | --- | --- | --- | --- |
|  |  | HBV RNA | HBcrAg | Anti-HBc | HDV RNA | HBV RNA | HBcrAg | Anti-HBc | HDV RNA |
| Baseline | HBcrAg | 0.399** |  |  |  | 0.361** |  |  |  |
|  | Anti-HBc | 0.300** | 0.183 |  |  | 0.391 | 0.137 |  |  |
|  | HDV RNA | 0161 | 0.327** | -0.154 |  | 0.110 | 0.289** | -0.204* |  |
|  | HBsAg | -0.016 | 0.519** | -0.017 | 0.641** | 0.071 | 0.517** | -0.074 | 0.402** |
| Follow-up 1 | HBcrAg | 0.429** |  |  |  | 0.353 |  |  |  |
|  | Anti-HBc | 0.362* | 0.136 |  |  | 0.279 | -0.041 |  |  |
|  | HDV RNA | 0.174 | 0.331* | -0.199 |  | 0.139 | 0.542** | -0.038 |  |
|  | HBsAg | 0.061 | 0.642** | -0.112 | 0.662** | 0.327 | 0.759** | 0.419 | 0.639** |
| Follow-up 2 | HBcrAg | 0.576** |  |  |  | 0.361 |  |  |  |
|  | Anti-HBc | 0.179 | 0.198 |  |  | 0.330 | -0.042 |  |  |
|  | HDV RNA | 0.077 | 0.377* | -0.162 |  | 0.504** | 0.617** | 0.012 |  |
|  | HBsAg | 0.005 | 0.493* | 0.074 | 0.742** | 0.309 | 0.854** | -0.189 | 0.749** |

Supplementary table 3. Correlation of virological parameters at baseline, after 6 months (follow-up 1) and 2-4 years (follow-up 2) of follow-up of patients with or without nucleos(t)ide analog (NA) treatment. Spearman correlation was used to calculate correlations. * p < 0.05 ** p < 0.001

# Supplementary table 4

|  | Development of the primary endpoint | | |
| --- | --- | --- | --- |
|  | No (44) | Yes (16) | p |
| Delta HBcrAg | 0.1 (0-0.38) | 0 (-0.1-0.18) | 0.141 |
| Delta anti-HBc | 174 (22-983) | 11 (-8-288) | 0.084 |
| Delta HDV RNA | 0.13 (-0.38-1.15) | 0.55 (-0.24-1.6) | 0.524 |
| HBcrAg decline | 7 (16) | 5 (31) | 0.273 |
| Anti-HBc decline | 7 (16) | 5 (31) | 0.273 |
| HDV RNA decline | 12 (27) | 5 (31) | 0.756 |

Supplementary table 4. Kinetics of virological parameters from baseline until follow-up 2 in patients with and without development of the primary (combined) endpoint. Patients with endpoints prior to follow-up 2 were excluded.

# Supplementary table 5

|  |  | | | Multivariable analyses: Model A | | | Multivariable analyses: Model B | | |
| --- | --- | --- | --- | --- | --- | --- | --- | --- | --- |
|  | Development of death/liver transplantation | | | HR | 95% CI | p-value | HR | 95% CI | p-value |
|  | No (151) | Yes (39) | p-value |  |  |  |  |  |  |
| Cirrhosis | 61 (40) | 37 (95) | **<0.001** | 15.51 | 3.67-65.60 | **<0.001** | 15.51 | 3.67-65.60 | **<0.001** |
| Male, n (%) | 97 (64) | 27 (69) | 0.559 |  |  |  |  |  |  |
| Age, years | 39.3 (30.5-47.6) | 49.5 (41.5-54.1) | **<0.001** | 1.04 | 1.01-1.08 | **0.018** | 1.04 | 1.01-1.08 | **0.018** |
| NA treatment | 65 (43) | 17 (44) | 0.951 |  |  |  |  |  |  |
| IFN prior to BL | 56 (37) | 11 (28) | 0.301 |  |  |  |  |  |  |
| HBV RNA detectable ^#^ | 18 (13) | 2 (6) | 0.537 |  |  |  |  |  |  |
| HBcrAg (log_10_ U/ml) | 3.9 (2.8-4.8) | 3.9 (3.0-4.6) | 0.824 |  |  |  |  |  |  |
| HBcrAg detectable | 111 (76) | 30 (77) | 0.664 |  |  |  |  |  |  |
| Anti-HBc (IU/ml) | 505 (158-1979) | 171 (51-709) | **0.005** | 1.0 | 1.0-1.0 | 0.310 |  |  |  |
| HBcrAg/anti-HBc ratio | 1.42 (1.02-1.95) | 1.63 (1.28-2.19) | **0.036** |  |  |  | 1.08 | 0.78-1.49 | 0.660 |
| HBsAg (IU/ml)^§^ | 8712 (2245-14116) | 2830 (2113-11412) | 0.180 |  |  |  |  |  |  |
| HDV RNA (log_10_ IU/ml) | 4.29 (2.0-5.64) | 4.71 (3.1-5.42) | 0.672 |  |  |  |  |  |  |
| HDV RNA detectable | 124 (82) | 35 (90) | 0.251 |  |  |  |  |  |  |

Supplementary table 5. Comparison of baseline characteristics of patients with and without the development of death/liver transplantation during follow-up. Continuous parameters are depicted as median with interquartile range, categorical variables as number with percentage. Mann Whitney U test, Chi-Square or Fisher’s exact test were used for group comparison. Cox-regression was used to perform multivariable analyses

# available for 143 and 32, respectively § available for 111 and 21, respectively

Abbreviations: CI, confidence interval; HR, hazard ratio; IFN, interferon; NA, nucleos(t)ide analog

# Supplementary table 6

|  | Development of hepatic decompensation/HCC | | | Multivariable analysis: Model A | | | Multivariable analysis: Model B | | |
| --- | --- | --- | --- | --- | --- | --- | --- | --- | --- |
| Variables | No (n=135) | Yes (n=55) | p-value | HR | 95% CI | p-value | HR | 95% CI | p-value |
| Male, n (%) | 83 (62) | 41 (75) | 0.086 |  |  |  |  |  |  |
| Age, years | 38.3 (29.2-45) | 50.5 (42.4-57.3) | **<0.001** | 1.063 | 1.032-1.095 | **<0.001** | 1.065 | 1.033-1.099 | **<0.001** |
| Cirrhosis, n (%) | 49 (36) | 49 (89) | **<0.001** | 5.554 | 2.442-12.63 | **<0.001** | 5.516 | 2.475-12.29 | **<0.001** |
| NA treatment | 52 (39) | 30 (55) | **0.043** | 1.233 | 0.678-2.241 | 0.490 | 1.178 | 0.6493-2.137 | 0.590 |
| IFN prior to BL | 50 (37) | 17 (31) | 0.423 |  |  |  |  |  |  |
| HBV RNA detectable | 17 (14) | 3 (6) | 0.169 |  |  |  |  |  |  |
| HBcrAg (log_10_ U/ml) | 3.8 (2.6-4.7) | 4.0 (3.1-4.8) | 0.309 |  |  |  |  |  |  |
| HBcrAg detectable | 97 (72) | 44 (80) | 0.244 |  |  |  |  |  |  |
| Anti-HBc (IU/ml) | 525 (143-2045) | 275 (82-709) | **0.005** |  |  |  | 1 | 1-1 | 0.08 |
| HBcrAg/anti-HBc ratio | 1.41 (0.97-1.87) | 1.72 (1.24-2.31) | **0.005** | 1.015 | 0.7923-1.301 | 0.910 |  |  |  |
| HBsAg (IU/ml) | 8627 (2062-14032) | 7016 (2409-13777) | 0.732 |  |  |  |  |  |  |
| HDV RNA (log_10_ IU/ml) | 4.22 (1.38-5.43) | 4.87 (2.98-5.57) | 0.254 |  |  |  |  |  |  |
| HDV RNA detectable | 109 (81) | 50 (91) | 0.085 |  |  |  |  |  |  |

Supplementary table 6. Comparison of baseline characteristics of patients with and without the development of hepatic decompensation or HCC during follow-up. Continuous parameters are depicted as median with interquartile range, categorical variables as number with percentage. Mann Whitney U test, Chi-Square or Fisher’s exact test were used for group comparison. Multivariable analysis of baseline variables for the development of hepatic decompensation or HCC was performed by using the Fine-Gray proportional hazard regression model for competing events with death/liver transplantation as competing events.

Abbreviations: BL, baseline; CI, confidence interval; HCC; hepatocellular carcinoma; HR, hazard ratio; IFN, interferon; NA, nucleos(t)ide analog

# Supplementary table 7

|  | HCC development | | |
| --- | --- | --- | --- |
|  | No (167) | Yes (23) | p-value |
| Cirrhosis | 79 (47) | 19 (83) | **<0.001** |
| Male, n (%) | 106 (64) | 18 (78) | 0.163 |
| Age, years | 39.9 (31.2-47.8) | 52.4 (45.8-59.5) | **<0.001** |
| NA treatment | 68 (41) | 14 (61) | 0.067 |
| IFN prior to BL | 58 (35) | 9 (39) | 0.679 |
| HBV RNA detectable ^#^ | 19 (13) | 1 (4) | 0.479 |
| HBcrAg (log_10_ U/ml) | 3.9 (2.9-4.7) | 4.2 (2.9-5.0) | 0.491 |
| HBcrAg detectable | 124 (74) | 17 (74) | 0.972 |
| Anti-HBc (IU/ml) | 474 (124-1660) | 388 (108-709) | 0.541 |
| HBcrAg/anti-HBc ratio | 1.44 (1.08-1.95) | 1.53 (1.03-2.19) | 0.437 |
| HBsAg (IU/ml)^§^ | 8824 (2423-14160) | 5625 (889-11768) | 0.090 |
| HDV RNA (log_10_ IU/ml) | 4.34 (2.60-5.43) | 4.34 (2.0-5.72) | 0.770 |
| HDV RNA detectable | 139 (83) | 20 (87) | 1.0 |

Supplementary table 7. Comparison of baseline characteristics of patients with and without the development of hepatocellular carcinoma during follow-up. Continuous parameters are depicted as median with interquartile range, categorical variables as number with percentage. Mann Whitney U test, Chi-Square or Fisher’s exact test were used for group comparison.

# available for 152 and 23, respectively § available for 114 and 18, respectively

Abbreviations: IFN, interferon; NA, nucleos(t)ide analog

# Supplementary table 8

|  | HBsAg loss during follow-up | | |
| --- | --- | --- | --- |
|  | No (181) | Yes (9) | p-value |
| Cirrhosis | 94 (52) | 4 (44) | 0.741 |
| Male, n (%) | 117 (65) | 7 (78) | 0.500 |
| Age, years | 41.2 (32.6-50) | 42 (34.6-48.6) | 0.845 |
| NA treatment, n (%) | 80 (44) | 2 (22) | 0.304 |
| IFN prior to BL, n (%) | 67 (37) | 0 (0) | **0.028** |
| HBV RNA detectable ^#^ | 20 (12) | 0 (0) | 0.600 |
| HBcrAg (log_10_ U/ml) | 4.0 (3.0-4.8) | 2.7 (2.0-3.2) | **0.011** |
| HBcrAg detectable | 138 (76) | 3 (33) | 0.010 |
| Anti-HBc (IU/ml) | 415 (113-1574) | 959 (449-1803) | 0.160 |
| HBcrAg/anti-HBc ratio | 1.45 (1.11-2.03) | 0.82 (0.70-1.30) | **0.003** |
| HBsAg (IU/ml)^§^ | 8712 (2570-14083) | 1594 (846-2886) | **0.020** |
| HDV RNA (log_10_ IU/ml) | 4.6 (2.78-5.61) | 1.66 (0.57-3.47) | **0.007** |
| HDV RNA detectable, n (%) | 152 (84) | 7 (78) | 0.642 |

Supplementary table 8. Comparison of baseline characteristics of patients with and without the HBsAg-loss during follow-up. Continuous parameters are depicted as median with interquartile range, categorical variables as number with percentage. Mann Whitney U test, Chi-Square or Fisher’s exact test were used for group comparison.

# available for 166 and 9, respectively § available for 127 and 5, respectively

Abbreviations: IFN, interferon; NA, nucleos(t)ide analog

# Supplementary table 9

|  | Development of the combined endpoint | | | | Multivariable analysis: Model A | | | | Multivariable analysis: Model B | | | |
| --- | --- | --- | --- | --- | --- | --- | --- | --- | --- | --- | --- | --- |
|  | No (102) | Yes (57) | p | HR | | 95% CI | p-value | HR | | 95% CI | p-value |  |
| Male, n (%) | 61 (59.8) | 40 (70.2) | 0.193 |  | |  |  |  | |  |  |  |
| Age, years | 37.7 (29.1-44.6) | 49.7 (41.2-55.1) | **<0.001** | 1.06 | | 1.030-1.091 | **<0.001** | 1.058 | | 1.028-1.089 | **<0.001** |  |
| Cirrhosis, n (%) | 34 (33.3) | 51 (89.5) | **<0.001** | 6.325 | | 2.643-15.13 | **<0.001** | 6.857 | | 2.884-16.3 | **<0.001** |  |
| NA treatment | 38 (37.3) | 29 (50.9) | 0.095 |  | |  |  |  | |  |  |  |
| IFN prior to BL | 39 (38.2) | 17 (29.8) | 0.287 |  | |  |  |  | |  |  |  |
| HBV RNA detectable ^#^ | 15 (15.6) | 3 (5.9) | 0.835 |  | |  |  |  | |  |  |  |
| HBcrAg (log_10_ U/ml) | 4.2 (3.28-4.8) | 4.3 (3.1-4.85) | 0.931 |  | |  |  |  | |  |  |  |
| HBcrAg detectable | 82 (80.4) | 45 (78.9) | 0.828 |  | |  |  |  | |  |  |  |
| Anti-HBc (IU/ml) | 553 (156-8-1786.8) | 214 (47.5-609.6) | **<0.001** | 0.999 | | 0.999-1.0 | 0.351 |  | |  |  |  |
| HBcrAg/anti-HBc ratio | 1.45 (1.14-1.94) | 1.83 (1.28-2.36) | **0.012** |  | |  |  | 1.123 | | 0.8645-1.459 | 0.3851 |  |
| HBsAg (IU/ml)^§^ | 10010 (5765-15857) | 9782 (2409-13777) | 0.077 |  | |  |  |  | |  |  |  |
| HDV RNA (log_10_ IU/ml) | 4.7 (3.58-5.86) | 4.91 (3.73-5.60) | 0.959 |  | |  |  |  | |  |  |  |

Supplementary table 9. Uni- and multivariate analysis of baseline characteristics of patients with detectable HDV RNA and with and without the development of the combined endpoint (decompensation, HCC, LTx/death) during follow-up. Continuous parameters are depicted as median with interquartile range, categorical variables as number with percentage. Mann Whitney U test, Chi-Square or Fisher’s exact test were used for group comparison. Multivariable Cox regression was used to address independent association of variables with the development of the combined endpoint during follow-up

# available for 147, § available for 107

Abbreviations: CI, confidence interval; HR, hazard ratio; IFN, interferon; NA, nucleos(t)ide analog

# Supplementary table 10

|  | Development of the combined endpoint | | |
| --- | --- | --- | --- |
|  | No (26) | Yes (5) | p |
| Male, n (%) | 18 (69.2) | 5 (100) | 0.291 |
| Age, years | 38.8 (30.1-45) | 58.1 (46-63.3) | **0.006** |
| Cirrhosis, n (%) | 8 (30.8) | 5 (100) | **0.008** |
| NA treatment | 12 (46.2) | 3 (60) | 0.654 |
| IFN prior to BL | 8 (30.8) | 3 (60) | 0.317 |
| HBV RNA detectable ^#^ | 2 (8.3) | 0 (0) | 1.0 |
| HBcrAg (log_10_ U/ml) | 2.0 (2.0-3.65) | 3.3 (2.9-3.75) | 0.193 |
| HBcrAg detectable | 11 (42.3) | 3 (60) | 0.636 |
| Anti-HBc (IU/ml) | 938 (250-4783) | 709 (140-21507) | 0.891 |
| HBcrAg/anti-HBc ratio | 0.87 (0.73-1.27) | 1.16 (0.95-1.36) | 0.354 |
| HBsAg (IU/ml)^§^ | 927 (86-2117) | 1689 (279-5154) | 0.446 |

Supplementary table 10. Univariate analysis of baseline characteristics of patients with undetectable HDV RNA and with and without the development of the combined endpoint (decompensation, HCC, LTx/death) during follow-up. Continuous parameters are depicted as median with interquartile range, categorical variables as number with percentage. Mann Whitney U test, Chi-Square or Fisher’s exact test were used for group comparison.

# available for 28, § available for 25

Abbreviations: CI, confidence interval; HR, hazard ratio; IFN, interferon; NA, nucleos(t)ide analog

# Supplementary table 11

|  | No cirrhosis (n=92) | Cirrhosis (n=98) | p-value |
| --- | --- | --- | --- |
| Male, n (%) | 55 (60) | 69 (70) | 0.1242 |
| Age, years | 36.3 (27.6-42.9) | 46.2 (38-52.4) | **<0.001** |
| NA treatment, n (%) | 33 (36) | 49 (50) | **0.049** |
| IFN prior to BL | 35 (38) | 32 (33) | 0.4371 |
| HBV RNA detectable, n (%) | 15 (18) | 5 (6) | **0.0102** |
| HBcrAg (log_10_ U/ml) | 3.95 (2.6-4.8) | 3.8 (3.0-4.6) | 0.9736 |
| HBcrAg detectable, n (%) | 65 (71) | 76 (78) | 0.2773 |
| Anti-HBc (IU/ml) | 687 (188-3388) | 309 (82-924) | **0.0004** |
| HBcrAg/anti-HBc ratio | 1.38 (0.97-1.85) | 1.55 (1.19-2.15) | **0.0157** |
| HBsAg (IU/ml) | 8522 (2543-15124) | 7869 (2062-12123) | 0.2607 |
| HDV RNA (log_10_ IU/ml) | 4.63 (1.20-5.72) | 4.30 (2.89-5.43) | 0.6106 |
| HDV RNA detectable, n (%) | 74 (80) | 85 (87) | 0.2402 |
| Sodium mmol/L | 140 (138-141) | 140 (137-141) | 0.266 |
| Creatinine µmol/L | 68 (59-78) | 66 (57-75) | 0.437 |
| AST U/L | 56 (34-79) | 71 (48-100) | **0.002** |
| ALT U/L | 80 (38-143) | 57 (38-108) | 0.400 |
| gGT U/L | 28 (19-53) | 73 (36-149) | **<0.001** |
| AP U/L | 75 (64-97) | 114 (83-161) | **<0.001** |
| CHE kU/L | 7.79 (6.26-9.11) | 4.16 (3.08-5.77) | **<0.001** |
| Bilirubin mmol/L | 10 (7-15) | 17 (11-34) | **<0.001** |
| Albumin g/L | 42 (37-45) | 35 (31-40) | **<0.001** |
| Platelets x1000/µl | 177 (150.3-223) | 67 (48-108.3) | **<0.001** |
| INR | 1.08 (1.02-1.13) | 1.27 (1.14-1.46) | **<0.001** |

Supplementary table 11. Baseline characteristics of patients with and without liver cirrhosis at baseline. Continuous parameters are depicted as median with interquartile range, categorical variables as number with percentage. Mann Whitney U test, Chi-Square or Fisher’s exact test were used for group comparison.

Abbreviations: AP, alkaline phosphatase; AST, aspartate aminotransferase; ALT, alanine aminotransferase; BL, baseline; CHE, cholinesterase; GGT, γ-glutamyltransferase; IFN, interferon; INR, international normalized ratio; NA, nucleos(t)ide analog

# Supplementary table 12

|  | Development of the combined endpoint | |  |
| --- | --- | --- | --- |
|  | No (n=86) | Yes (n=6) | p-value |
| Male, n (%) | 50 (58) | 5 (83) | 0.396 |
| Age, years | 35.1 (27.6-42.5) | 41.5 (33.2-47.6) | **0.003** |
| NA treatment | 30 (35) | 3 (50) | 0.663 |
| IFN prior to BL | 33 (38) | 2 (33) | 1.0 |
| HBV RNA detectable^#^ | 14 (18) | 1 (20) | 1.0 |
| HBcrAg (log_10_ U/ml) | 3.9 (2.53-4.8) | 4.95 (3.2-5.83) | 0.211 |
| HBcrAg detectable | 60 (70) | 5 (83) | 0.667 |
| Anti-HBc (IU/ml) | 729 (195-3500) | 341 (75-1415) | 0.204 |
| HBcrAg/anti-HBc ratio | 1.35 (0.93-1.81) | 1.88 (1.29-2.66) | 0.077 |
| HBsAg (IU/ml)^§^ | 8522 (2543-15453) | 8790 (2518-13652) | 0.798 |
| HDV RNA (log_10_ IU/ml) | 4.32 (1.15-5.61) | 5.72 (5.34-5.86) | **0.025** |
| HDV RNA detectable | 68 (79) | 6 (100) | 0.593 |
| Sodium mmol/L | 140 (138-141) | 139 (138-142) | 0.775 |
| Creatinine µmol/L | 68 (59-78) | 72 (64-82) | 0.492 |
| AST U/L | 50 (34-80) | 70 (65-196) | 0.065 |
| ALT U/L | 70 (37-142) | 95 (74-314) | 0.207 |
| gGT U/L | 28 (19-49) | 67 (40-233) | **0.016** |
| AP U/L | 73 (64-98) | 92 (52-175) | 0.458 |
| CHE kU/L | 7.84 (6.3-9.33) | 6.49 (5.29-7.49) | 0.119 |
| Bilirubin mmol/L | 10 (7-15) | 7 (5.5-14) | 0.390 |
| Albumin g/L | 42 (37-46) | 46 (34-39) | **0.024** |
| Platelets x1000/µl | 181 (156.5-224.3) | 99.5 (88.3-147.3) | **<0.001** |
| INR | 1.08 (1.02-1.12) | 1.14 (1.12-1.19) | **0.030** |

Supplementary table 12. Comparison of baseline characteristics of patients without liver cirrhosis with and without the development of the combined endpoint during follow-up. Continuous parameters are depicted as median with interquartile range, categorical variables as number with percentage. Mann Whitney U test, Chi-Square or Fisher’s exact test were used for group comparison.

# available for 84 samples

§ available for 64 samples

Abbreviations: AP, alkaline phosphatase; AST, aspartate aminotransferase; ALT, alanine aminotransferase; BL, baseline; CHE, cholinesterase; GGT, γ-glutamyltransferase; IFN, interferon; INR, international normalized ratio; NA, nucleos(t)ide analog

# Supplementary figures

## Supplementary figure 1

Correlation of HBcrAg and HBV RNA (A), HBcrAg and HDV RNA (B), HBcrAg and HBsAg (C), and HBsAg and HDV RNA (D).

## Supplementary figure 2

Correlation of HBcrAg and HBV RNA in samples with detectable HBV RNA.
